# A Novel Method for Identifying Crack and Shaft Misalignment Faults in Rotor Systems under Noisy Environments Based on CNN

**DOI:** 10.3390/s19235158

**Published:** 2019-11-25

**Authors:** Wang Zhao, Chunrong Hua, Dawei Dong, Huajiang Ouyang

**Affiliations:** 1 School of Mechanical Engineering, Southwest Jiaotong University, Chengdu 610031, China; zw309844359@163.com (W.Z.); dwdong@swjtu.cn (D.D.); 2 School of Engineering, University of Liverpool, Liverpool L69 3GH, UK; h.ouyang@liverpool.ac.uk

**Keywords:** crack, shaft misalignment, fault identification, convolutional neural network, noisy environment

## Abstract

Crack and shaft misalignment are two common types of fault in a rotor system, both of which have very similar dynamic response characteristics, and the vibration signals are vulnerable to noise contamination because of the interaction among different components of rotating machinery in the actual industrial environment, resulting in great difficulties in fault identification of a rotor system based on vibration signals. A method for identification of faults in the form of crack and shaft misalignments is proposed in this paper, which combines variational mode decomposition (VMD) and probabilistic principal component analysis (PPCA) to denoise the collected vibration signals from a test rig and then achieve signal feature extraction and fault classification with convolutional artificial neural network (CNN). The key parameters of the CNN are optimized and determined by genetic algorithm (GA) firstly, and the domain adaptability of the trained network is verified by the signals with different signal-to-noise ratio (SNR) values; then, the noisy vibration signals are decomposed into multiple band-limited intrinsic modal functions by VMD, and further data dimension reduction is performed by PPCA to realize the separation of the useful signals from noise; finally, the crack and shaft misalignment of the rotor system are identified by the optimized CNN. The results show that the proposed method can effectively remove the interference noise and extract the intrinsic features of the vibration signals, and the recognition rates of crack and shaft misalignment faults for the rotor system with different SNR values are more than 99%, which is considered to be very effective and useful.

## 1. Introduction

As the core component of rotating machinery, the health status of a rotor system will directly affect the normal operation of the entire equipment; however, as a rotor system often operates in a high temperature and high pressure environment, stress concentration will appear in some parts of the rotating shaft and fatigue cracks may gradually occur after long operation times, due to the long-term effect of complex alternating loads at high speed. The crack propagation speed will increase sharply when the crack reaches a certain depth, and the rotating shaft is likely to fracture in a very short time, which will not only cause the outage of mechanical equipment, resulting in immeasurable economic losses, but also may lead to catastrophic accidents, threatening the personal safety of the personnel involved. Therefore, the serious loss of life and property can be avoided if structural health monitoring of a rotor system can be performed to detect the crack fault in time.

Most faults are caused by the mechanical vibration of rotating equipment, and the vibration signal of the object is often easy to acquire, thus the fault diagnosis method based on vibration signals has been widely used, which can intuitively and accurately characterize the dynamics of equipment, and has the advantages of simplicity, practicability, and low cost. In [1,2], the influence of parameter variation of a transverse crack on the inherent characteristics of a rotor system was studied by means of both simulation and experiment, and the crack was identified. Rahman et al. [3] proposed a method for detecting the crack in a static rotor using the phase angle change in frequency response function. Tlaisi et al. [4] performed crack detection on a cracked rotor with a propeller by testing the change rate of the torsional and bending natural frequencies. Ishida et al. [5] studied the nonlinear response characteristics of a breathing-cracked rotor under harmonic excitation, and concluded that the combination of rotation and excitation could be used for crack detection. Mani et al. [6] found that an appropriate excitation frequency could stimulate the combination resonance of rotational and natural frequencies, and the vibration amplitude corresponding to the natural frequency was proportional to the crack depth, and the crack was thus quantified; in addition, the authors further performed a time-frequency analysis on the response of combination resonance by continuous wavelet transform (CWT), and improved the crack identification accuracy in rotor systems [7].

Chasalevris et al. [8] realized online crack detection by testing the additional lateral coupled vibration response generated by a cracked rotor under external excitation force. Sinha [9,10] used the characteristics of high-harmonic signal components generated by a fatigue crack to it in a rotor. Guo et al. [11] and Babu et al. [12] used Hilbert–Huang transform (HHT) to analyze the transient response of a cracked rotor at critical speed in startup condition, and concluded that HHT was better than FFT and CWT in crack detection for an unsteady rotor. Nagaraju et al. [13] introduced phase information based on a traditional wavelet transform and applied it to crack detection for unsteady rotors. Jesus et al. [14] trained an artificial neural network with energy characteristics of vibration signals obtained by wavelet packet transformation (WPT), and realized crack identification in a rotor at different speeds. Saridakis [15] established a relationship between the rotation angle and the flexibility coefficient of a crack element through an artificial neural network for a rotor system with two transverse cracks, built the objective function of the natural frequency and vibration response based on fuzzy logic, and finally achieved crack detection. In summary, the fault diagnosis method based on vibration signal can achieve crack detection in rotor systems to a certain extent, which is sensitive to fault features and convenient for testing. However, the identification accuracy often depends on different signal processing methods and an expert’s diagnosis experience, and the process of feature extraction is time-consuming. Therefore, how to expedite the signal analysis procedure and realize the feature extraction automatically is the key to achieving intelligent fault diagnosis of rotor systems.

Some researchers found that the 2× superharmonic component could be used as the characteristic of a crack fault for a rotor system [16], and the 2× and 3× superharmonic components of the cracked rotor system are particularly prominent, especially at 1/2 and 1/3 subcritical speeds [17]. In [18,19,20,21], the presence of cracks was successfully detected by analyzing the 2× and 3× superharmonic components of rotor vibration signals. In practice, the machining errors of couplings and the uneven thermal deformation of each part of a rotor often causes shaft misalignment, which is also a very common fault in a rotor system, and the corresponding dynamic responses also produce 2× superharmonic components similar to that by a crack fault, easily resulting in fault misdiagnosis for a rotor system [22]. Up to now, there are few studies on the identification of both crack and shaft misalignment of a rotor system. Sekhar et al. [23] extracted the subharmonic resonance peak of vibration signals of a rotor shaft with CWT in speed-up condition, and realized the distinction between crack and shaft misalignment, but could not achieve the fault classification of the rotor in steady state. Patel and Darpe [24] modeled a rotor system with six-degrees-of-freedom Timoshenko beam elements, and studied the influence of shaft misalignment on the steady-state vibration response at subcritical speeds, and the faults of crack and shaft misalignment were distinguished by a full spectrum analysis and the rotor axis orbit, but the method failed to identify the misalignment–crack coupling faults. As cracked and misaligned rotors can both produce superharmonic components in vibration signals, it is difficult to distinguish them accurately relying on only the vibration characteristics in frequency domain. Moreover, the coupling of crack and shaft misalignment in rotor systems will produce more complex nonlinear dynamic characteristics in vibration responses and greatly increase the difficulty of fault identification for rotor systems.

In addition, in the actual industrial environment, the vibration signals of the rotating machinery will inevitably encounter noise interference, and how to automatically extract the fault characteristic information of vibration signal under different noisy environments brings great challenges to the fault diagnosis method for rotor systems based on vibration signals.

In this paper, a method for identifying crack and shaft misalignment in noisy environments is proposed by combining VMD, PPCA, and CNN. Firstly, the key parameters of a CNN are optimized and determined by GA and, meanwhile, the optimized network is trained by the original measured signals in the laboratory, and the domain adaptability of the network is verified by the noisy signals with different SNR values. Secondly, the noisy signals are decomposed into multiple band-limited intrinsic modal functions by VMD, and further data dimension reduction is performed by PPCA to realize the separation of useful signals from noise. Finally, the optimized CNN is used to automatically extract the characteristic information of the denoised signals and achieve multifault diagnosis. The vibration signals of the rotor system under different working conditions are measured on a test rig, and the Gaussian white noise with different SNR values are added to simulate the noisy signals in industrial environment, and the identification of crack and shaft misalignment is eventually carried out, effectively, by the proposed method.

## 2. Introduction of Convolutional Neural Network

Convolutional neural network is a multilayer supervised learning neural network. The lower hidden layers of the CNN are composed of the convolutional layers and the pooling layers alternately which are the core modules for feature extraction, while the upper layers are the fully connected layers and the logistic regression classifier. The recently proposed batch normalization layer is often used after the convolutional layer to speed up network convergence and avoid vanishing gradients [25], and it is then necessary to process data through an activation function which can nonlinearly express the extracted features. Moreover, the gradient descent method is adopted to minimize the loss function to adjust the weight parameters in network layer by layer, and the accuracy of the network is improved through frequent iterative training. 

### 2.1. Convolutional Layer

The convolutional layer highlights two major characteristics of a CNN, namely sparse connection and weight sharing. On the one hand, the neuron nodes of each layer are only connected to the upper local neuron nodes, which greatly reduces the parameter size of the network. On the other hand, each kernel of the convolution layer is repeatedly applied to the whole receptive field to convolve the input data, and each kernel shares the same weights and biases to improve the training speed. The specific convolution process is

(1) $$x_{j}^{l}=f\left(\sum_{i}x_{i}^{l-1}*w_{ij}^{l}+b_{j}^{l}\right)$$

where $x_{j}^{l}$ denotes the *j*th local region in layer *l*, $w_{ij}^{l}$ denotes the weight between the *j*th kernel and the *i*th input local region, $b_{j}^{l}$ denotes the bias of the *j*th kernel, the notation ∗ denotes the convolution calculation, and f(⋅) denotes the nonlinear activation function.

### 2.2. Batch Normalization Layer

A batch normalization layer, which can obviously accelerate the training speed and improve the generalization capability of the network, is generally applied to normalize the output data after the convolutional layer so that each block of the network maintains the same distribution before inputting to the activation layer. The transformation process of a batch normalization layer is as follows:

(2) $$\begin{array}{l} {\hat{x}}^{\left(k\right)}=\frac{x^{\left(k\right)}-E[x^{\left(k\right)}]}{\sqrt{\mathrm{Var}[x^{\left(k\right)}]}}\\ y^{\left(k\right)}=\gamma^{\left(k\right)}{\hat{x}}^{\left(k\right)}+\beta^{\left(k\right)} \end{array}$$

where $E[x^{\left(k\right)}]$ is the mean value of each batch of training data $x^{\left(k\right)}$, $\mathrm{Var}[x^{\left(k\right)}]$ is the variance of each batch of data, $y^{\left(k\right)}$ is the output of neurons, and $\gamma^{\left(k\right)}$ and $\beta^{\left(k\right)}$ are the scale and shift parameters in transformation reconstruction, respectively.

### 2.3. Pooling Layer

The convolutional layer is usually followed by a pooling layer whose function is to extract the local mean value or maximum value. According to different calculated values, it can be divided into average pooling layer and max pooling layer. The pooling process can significantly reduce the computational complexity of the upper layer while retaining useful information, thus effectively reducing the risk of overfitting. The pooling operation is defined as

(3) $$x_{j}^{l}=\mathrm{down}\left(x_{j}^{l-1},s\right)$$

where down(⋅) represents the down-sampling function, $x_{j}^{l-1}$ represents the *j*th local region in layer *l −* 1, and *s* represents the pooling block size.

### 2.4. Fully Connected Layer

Unlike the convolutional layer, the neuron nodes of the fully connected layer are connected to all the upper nodes. The fully connected layer is usually applied to the end of a CNN to map the learned feature representation into the sample space, and the output data will be passed to the final classifier, typically a softmax function. Aimed at an *n*-label classification task, the specific calculation process of the softmax function is as follows:

(4) $$P_{n}=\frac{\exp\left(x_{i}\right)}{\sum_{j=1}^{m}\exp\left(x_{j}\right)}$$

where $P_{n}$ is the probability of the input $x_{i}$ belonging to label *n*.

## 3. The Proposed Intelligent Diagnosis Method

Focusing on the difficulty in distinguishing the crack and shaft misalignment in a rotor system in a noisy environment, the fault identification process is divided into two cases, namely, the raw training samples and training samples with added noise.

(1) When the training samples are original measured signals, the GA is used to optimize the main structural parameters of the CNN, and the batch size and learning rate of which are adjusted to adapt to the dataset. The raw time-domain signals are directly used as the input of the optimized CNN, then the noisy signals with different SNR values are used to verify the performance of the CNN model.
(2) In the actual industrial environment, the complicated structure of large-scale mechanical equipment and the interaction between different components make it difficult to obtain the original vibration signals, and in the absence of a sample database, it is necessary to use the noisy signals in network training; on the other hand, the vibration signals measured on a test rig are invulnerable to noise, while signals in engineering have relatively low SNR values. In this paper, vibration signals in an actual noisy environment are simulated by adding noise to the vibration signals measured on a test rig, and a fault identification method combining VMD, PPCA, and CNN is proposed to achieve high-precision identification of different types of faults for a rotor system in a noisy environment.

### 3.1. Parameter Optimization of CNN Based on GA

**Figure 1:**
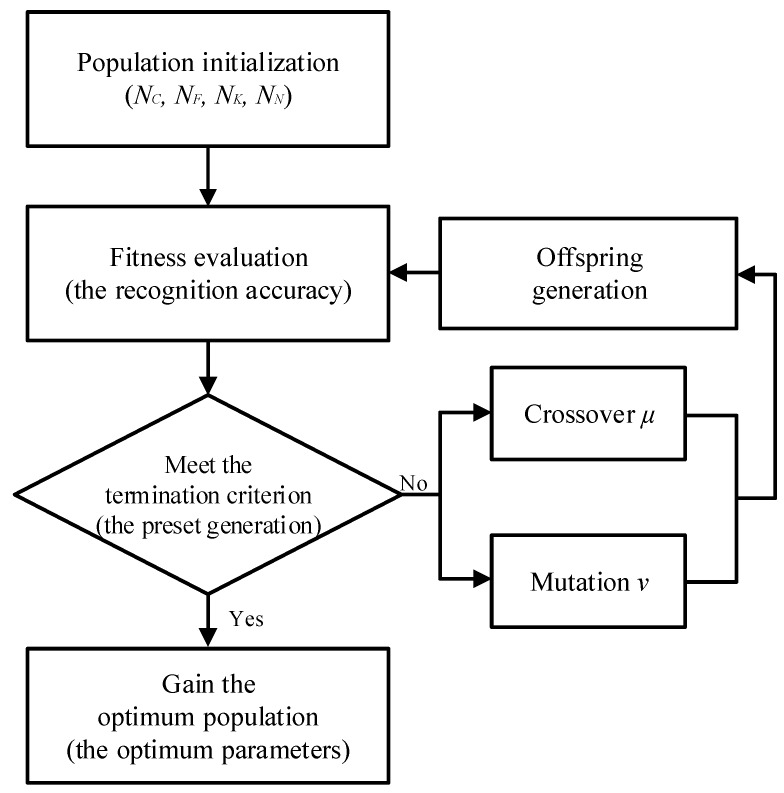
The flowchart of genetic algorithm.

For different tasks and datasets, a CNN often requires artificial and repeated experiments to design different structural parameters, which is time-consuming, laborious, and relies on historical experience. In this paper, GA is utilized to optimize the network parameters of a CNN adaptively, which is a global search algorithm inspired by evolutionism in biology and widely used in multiobjective optimization problems. The numbers of convolutional layers $N_{C}$ of fully connected layers $N_{F}$ of convolutional kernels $N_{K}$ and of nodes in fully connected layers $N_{N}$ should be determined firstly when designing the structure of a CNN, and the selection of different parameters will directly affect the final classification results. GA is used to determine parameters $N_{C}$, $N_{F}$, $N_{K}$, and $N_{N}$ as the population to be optimized to avoid a large number of empirical choices, and the parameters of each group are encoded as chromosomes. Different combinations of network parameters are generated by crossover and mutation of chromosomes, and the fitness, i.e., the classification accuracy of CNN under each parameter combination mode is calculated, and then the optimal population, i.e., the optimal network parameter configurations are determined. The flowchart of parameter optimization for the CNN is shown in Figure 1.

### 3.2. VMD

As an adaptive and nonrecursive signal decomposition method, VMD, has a solid theoretical foundation and the essence is in the construction and solution of variational problems [26]. It can adaptively decompose any complex multicomponent signal into a series of band-limited intrinsic modal function with different central frequencies and limited bandwidths, which can effectively suppress the endpoint effect and have a good effect on the processing of nonstationary signals.

In VMD, intrinsic modal functions (IMF) are defined as a series of AM–FM signals whose expressions are as follows:

(5) $$u_{k}(t)=A_{k}(t)\cos(\varphi_{k}(t))$$

where $A_{k}(t)$ and $\varphi_{k}(t)$ denote the functions of amplitude and phase varying with time, respectively. Assuming that the multicomponent signal f(t) is composed of the *k* IMF component $u_{k}(t)$ of finite bandwidth, $\omega_{k}$ is the central frequency of each IMF component, establishing the constrained variational model as

(6) $$\begin{array}{l} \underset{\left\{u_{k}\},\{\omega_{k}\right\}}{\min}\left\{\sum_{k=1}^{K}{\left\|\partial_{t}[(\delta (t)+\frac{j}{\pi t})*u_{k}(t)]e^{-j\omega_{k}t}\right\|}_{2}^{2}\right\}\\ s.t.\sum_{k}u_{k}(t)=f(t) \end{array}$$

where δ(t) is the impulse function and ∗ is the convolution operator.

The quadratic penalty term and Lagrangian multiplier are introduced to render the problem unconstrained to convert the above constrained variational problem into

(7) $$L(\{u_{k}\},\{\omega_{k}\},\lambda )=\alpha {\sum_{k=1}^{K}\left\|\partial_{t}\left[\left(\delta (t)+\frac{j}{\pi t}\right)*u_{k}(t)\right]e^{-j\omega_{k}t}\right\|}_{2}^{2}+{\left\|f(t)-\sum_{k=1}^{K}u_{k}(t)\right\|}_{2}^{2}+\langle \lambda (t),f(t)-\sum_{k=1}^{K}u_{k}(t)\rangle$$

where α is the penalty factor and λ is the Lagrangian multiplier. Equation (8) is then solved by the alternate direction method of multipliers (ADMM) [27], and the updated equation of mode $\mu_{k}(\omega )$ and central frequencies $\omega_{k}$ are shown in Equations (8) and (9), respectively.

(8) $${\hat{u}}_{k}^{n+1}(\omega )=\frac{\hat{f}(\omega )-\sum_{i\neq k}{\hat{u}}_{i}(\omega )+\frac{\hat{\lambda }(\omega )}{2}}{1+2\alpha {\left(\omega -\omega_{k}\right)}^{2}}$$

(9) $$\omega_{k}^{n+1}=\frac{\int_{0}^{\infty }\omega |{\hat{u}}_{k}(\omega ){|}^{2}d\omega }{\int_{0}^{\infty }|{\hat{u}}_{k}(\omega ){|}^{2}d\omega }$$

where ${\hat{u}}_{k}(\omega )$, $\hat{f}(\omega )$, and $\hat{\lambda }(\omega )$ are the Fourier transformations of $u_{k}(t)$, f(t), and λ(t), respectively. The detailed steps and description of the VMD algorithm can be found in [26].

### 3.3. PPCA

PPCA is a latent variable model with factor analysis method, which is a probabilistic generalization of principal component analysis [28]. As a data dimensionality reduction method, PPCA needs to establish an appropriate probability model, and the main components and fault information of the raw signal are stored in the principal component subspace, while the noise and linear correlation information are discarded in the remaining subspace. The essence of PPCA is to take the direction of the maximum variance as the main feature, and dissociate the data in each orthogonal direction, that is, to make them uncorrelated in different orthogonal directions. Therefore, PPCA can not only remove the interference noise, but also enhance the retention of raw signal characteristic information, which has been widely used in the fields of feature extraction and pattern recognition.

Consider the following probability distribution model

(10) X=P⋅u+E

where $X=\left\{x_{1},x_{2},\ldots ,x_{m}\right\}\in R^{n\times m}$ is an n-by-m normalized matrix, n is the number of the original variables (embedding dimension), *m* is the number of samples; the n-by-k matrix $P=\left\{p_{1},p_{2},\ldots ,p_{k}\right\}\in R^{n\times k}$ is the loading matrix (principal component matrix) with the limiting condition of *k* < *n*, and *k* is the number of the principal components. The k-by-m matrix $u=\left\{u_{1},u_{2},\ldots ,u_{m}\right\}\in R^{k\times m}$ is the principal component matrix and is set to meet Gaussian distribution with zero mean and **I** (identity matrix) covariance, i.e., N(0,I). **E** is an isotropic Gaussian noise matrix and is set to satisfy $N\left(0,\sigma^{2}I\right)$, where $\sigma^{2}$ denotes the variance of noise variables. Therefore, **X** meets $N\left(0,PP^{T}+\sigma^{2}I\right)$

The probability distribution of **u** is given by

(11) $$p(u)={\left(2\pi \right)}^{-k/2}\exp\left\{-\frac{1}{2\sigma^{2}}X^{T}X\right\}.$$

The conditional probability distribution over **x**-space for a given **u** is

(12) $$p(x|u)={\left(2\pi \right)}^{-n/2}\exp\left\{-\frac{1}{2\sigma^{2}}{\left\|X-P\cdot u\right\|}^{2}\right\}.$$

Therefore, the probability distribution of **x** is

(13) $$p(x)=\int p(x|u)p(u)dx={\left(2\pi \right)}^{-n/2}{\left|C\right|}^{-1/2}\exp\left\{-\frac{1}{2}x^{T}C^{-1}x\right\}$$

where $C=PP^{T}+\sigma^{2}I$ is an n×n covariance matrix determined by **P** and $\sigma^{2}$

According to Bayes’ theorem [29,30], conditional probability distribution of **u** for a given **x** is

(14) $$p(u|x)={\left(2\pi \right)}^{-\frac{k}{2}}{\left|{\left(\sigma^{2}\right)}^{-1}M\right|}^{1/2}\exp\left\{-\frac{1}{2}{\left[u-M^{-1}P^{T}X^{T}\right]}^{T}\left({\left(\sigma^{2}\right)}^{-1}M\right)\left[u-M^{-1}P^{T}X^{T}\right]\right\}$$

where $M=P^{T}P+\sigma^{2}I$ is a k×k matrix whose dimensions have been reduced.

As shown in Equations (11)–(14), the probability model can be obtained once the parameters **P** and $\sigma^{2}$ are determined. The expectation–maximization (EM) algorithm [31,32,33] can be selected to estimate parameters as

(15) $$\begin{array}{l} \tilde{P}=SP(\sigma^{2}I+M^{-1}P^{T}SP{)}^{-1}\\ {\tilde{\sigma }}^{2}=\frac{1}{n}\mathrm{tr}\left(S-SPM^{-1}{\tilde{P}}^{T}\right) \end{array}$$

where $\tilde{P}$ and ${\tilde{\sigma }}^{2}$ are the corresponding updating parameters, $S=\frac{1}{m}\sum_{i=1}^{m}x_{i}x_{i}{}^{T}$ is the covariance matrix of the original variables, tr(⋅) represents the trace of matrix.

By repeatedly iterating Equation (15) until convergence, **P** and $\sigma^{2}$ are calculated, and the PPCA model is finally obtained.

### 3.4. General Procedure of the Proposed Method

**Figure 2:**
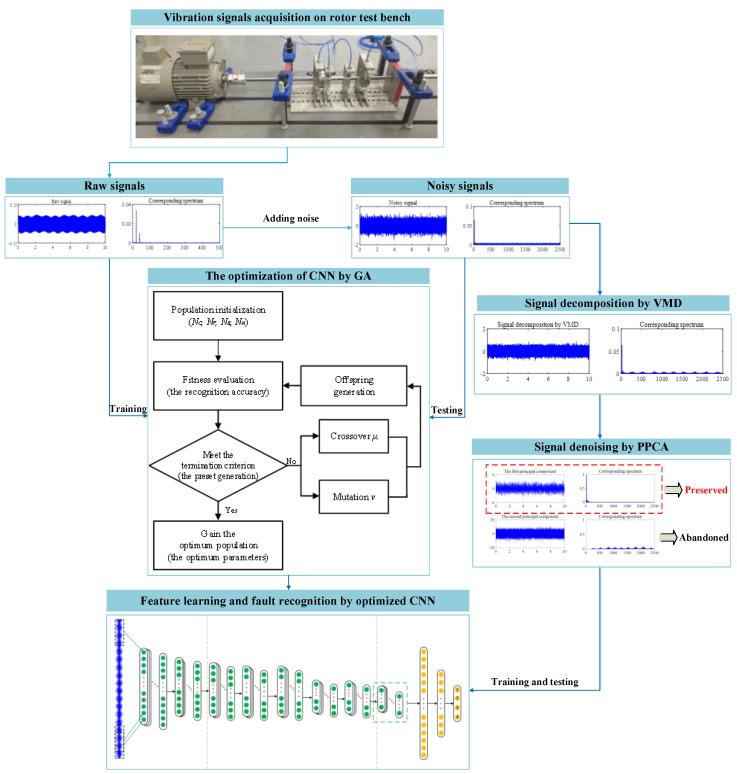
The framework of the proposed method.

In order to diagnose the faults of crack and shaft misalignment in a rotor system in different noisy environments, two cases are studied. The flowchart of the proposed method is given in Figure 2, and the main procedure is summarized as follows:

- Step 1: Different operating conditions of the rotor system are implemented in the test rig, and the vibration signals of the rotor system are collected by eddy current displacement sensors and a data acquisition system.
- Step 2: The collected raw time-domain signals are randomly divided into a training set and a testing set. The training set is directly input to a CNN for training, and the testing set is contaminated with Gaussian white noise to verify the performance of the CNN model. At the same time, the GA is used to optimize the numbers of convolutional layers, convolutional kernels, fully connected layers, and nodes in fully connected layers to obtain the best network structure parameters.
- Step 3: The batch size and learning rate are further determined to obtain the optimal network model.
- Step 4: Noises with different energy levels are added to the collected vibration signals, and the noisy signals are decomposed into *k* submodal functions with different frequency bands by VMD algorithm.
- Step 5: The decomposed k-dimensional signals are reduced to two-dimensional ones by PPCA to realize the separation of the denoised useful signals and the noise.
- Step 6: The denoised useful signals are taken as the input of the optimized CNN model, and the fault identification of crack and shaft misalignment for the rotor system in different noisy environments are realized through network learning.

## 4. Experimental Verification

Two experimental cases are considered respectively to verify the proposed method in noisy environments, where Case 1 is the fault diagnosis of the rotor system in which the training samples are raw measured signals and the testing samples are noisy signals, while Case 2 contains the training samples and the testing samples that are both noisy.

### 4.1. Experimental Setup

**Figure 3:**
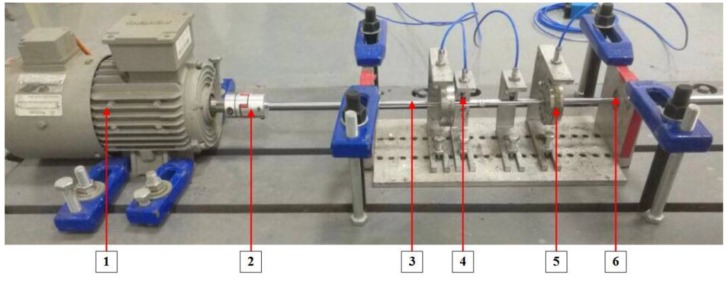
Illustration of the rotor test bench: (1) motor, (2) flexible coupling, (3) shaft, (4) eddy current displacement sensor, (5) rotary disc, (6) bearing.

To validate the effectiveness of the proposed method, we carried out the experiments on a rotor test bench. Figure 3 shows the structure of the test bench and the arrangement of the sensors, which composes a driving motor, a motor control system, a coupling, a rotor, two rotary tables, bearings, sensors, and a signal acquisition system, and the relevant parameters of each component are shown in Table 1. The vibration signals of the rotor system are measured by the four eddy current displacement sensors and acquired through the signal acquisition system. Three rotating speeds and four working conditions are particularly considered in this paper.

### 4.2. Data Description

There are four kinds of conditions of the rotor system: healthy, shaft misalignment fault, crack fault, and misalignment–crack coupling fault. In the rotor fault experiment, it is necessary to replace the rotating shaft with a crack, which is machined by wire cutting with a depth of 4 mm. In addition, it is necessary to simulate the misalignment fault by raising the support part of the motor by 0.1 mm through a gasket. Vibration signals of the rotor system at multiple rotating speeds are collected and the experiments are conducted as follows:

(1) Adjust the motor control system to a specific rotating speed and keep the speed for at least 60 s. Then, collect the vibration signals for 20 s by the data acquisition system with a sampling frequency of 5 kHz;
(2) Change the motor speed and repeat the vibration signal acquisition as mentioned above;
(3) Change the fault types of the rotor system and repeat the above experimental operations.

Considering the influence of rotating speed on fault classification, three groups of signals at rotating speeds of 10, 21, and 30 Hz are selected from the collected data to simulate the operating conditions of the rotor system at low, medium, and high speeds, respectively. Each group of signals is normalized and the data augment with overlap is adopted to form the sample sets. In detail, there are 1000 samples for each operating condition, and each sample contains 1024 data points; therefore, the final datasets contain a total of 4000 samples, of which 70% are randomly selected to form the training set and the rest are the testing set.

### 4.3. Network Construction of CNN

#### 4.3.1. Parameter Optimization Based on GA

When using GA to optimize the parameters of a CNN, the number of convolutional layers, kernels, fully connected layers, and nodes in the fully connected layers are mainly considered, and pooling layers and batch normalization layers are also needed for an integrated CNN structure. However, the computational complexity of the algorithm will be significantly increased if too many parameters are considered. The numbers of pooling layers and batch normalization layers are consistent with the number of convolutional layers (optimized) in common network design methods to avoid using too much computing time in the optimization process and ensure the effectiveness of the network, and the parameters of GA are shown in Table 2. The values of $N_{K}$ and $N_{N}$ are selected as the power exponents of 2 to prevent the slow calculation due to the large population size, and the optimized parameters are shown in Table 3.

**Figure 4:**
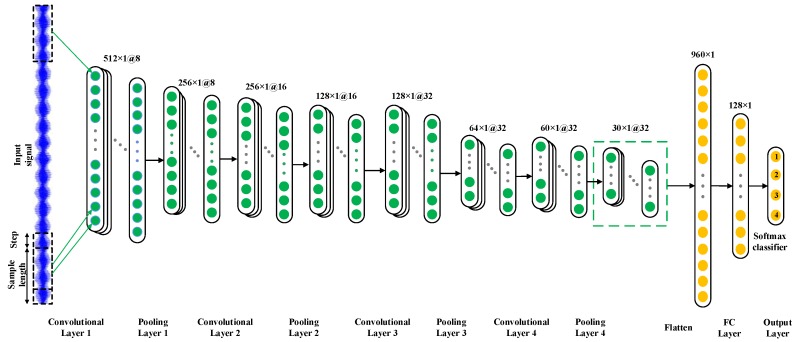
Architecture of the optimized model.

Moreover, in [34], it was pointed out that the selection of a wide kernel in the first convolutional layer of a CNN can extract the medium and low-frequency features of the signals and reduce the high-frequency noise to a certain extent, therefore a kernel of size 64 × 1 with a stride of size 4 × 1 is selected in the first convolutional layer in this paper. Moreover, the pooling type is max pooling and the activation function is ReLU, and the detailed architecture of the CNN optimized by GA is shown in Figure 4.

#### 4.3.2. Small Batch Size Training and Learning Rate Decay

In the process of network training, the batch size and learning rate are two main parameters that affect the accuracy and speed. A larger batch size will speed up the data processing and reduce the iterations needed for one epoch, but it may also cause the training process to fall into a local optimal solution, resulting in the decline in generalization ability of the model [35,36], and an increase in the epochs required for network convergence. A smaller batch size will introduce noise into the network learning, which has a certain regularization effect, but will also cause severe oscillation of loss function and convergence difficulty in the training process. For the learning rate, the larger the value, the faster the model training, but it may lead to difficulty in convergence; on the other hand, the smaller the value, the higher the training accuracy, but the longer the training time. Accordingly, this paper selects a small batch size of 16 and adopts learning rate decay to balance the convergence and training accuracy of the model and improve the generalization ability of the network. Moreover, stochastic gradient descent (SGD) with momentum algorithm is adopted with an initial learning rate of 0.01, and the momentum size defaults to 0.9. During the network training, the learning rate is 0.5 times the previous one after every 10 epochs, and the total number of epochs is 40. Ten trials are conducted on each dataset to avoid random interference.

### 4.4. Case 1: Fault Diagnosis for Noise-Free Training Samples

**Figure 5:**
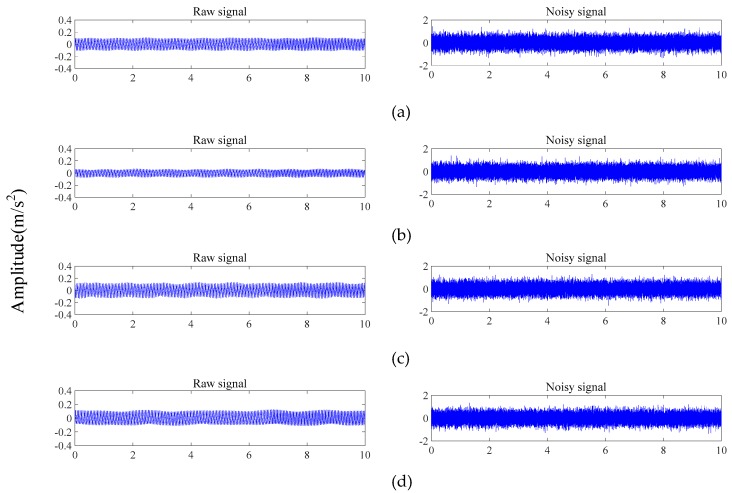
Raw signals and their corresponding noisy signals of the four conditions: (**a**) normal condition, (**b**) crack fault, (**c**) shaft misalignment fault, and (**d**) simultaneous misalignment–crack fault.

The collected raw time-domain signals are randomly divided into a training set and a testing set, and the training set is directly used as input for network training. Gaussian white noise with different SNR values are added to the testing set signals to simulate the different noise backgrounds in industrial environment, so as to verify the effectiveness and anti-interference ability of the network. The signal-to-noise ratio (SNR) is defined as

(16) $${\mathrm{SNR}=10\log}_{10}\left(\frac{P_{\mathrm{signal}}}{P_{\mathrm{noise}}}\right),$$

where $P_{\mathrm{signal}}$ and $P_{\mathrm{noise}}$ are the powers of the signal and the noise, respectively. The collected raw signals and corresponding noisy signals (SNR = 10 dB) of four conditions at 21 Hz are shown in Figure 5, and the classification results of the testing set with different SNR values are shown in Table 4.

It can be seen from Table 4 that the optimized CNN based on GA has realized high-precision identification of the test samples with different SNR values. Except for the relatively low accuracy corresponding to a strong noise background with an SNR of −10 dB, all of the accuracy values are close or equal to 100% when the SNR value is greater than −6 dB. Accordingly, the designed CNN based on GA has good domain adaptability and can effectively identify multiple faults of a rotor system.

**Figure 6:**
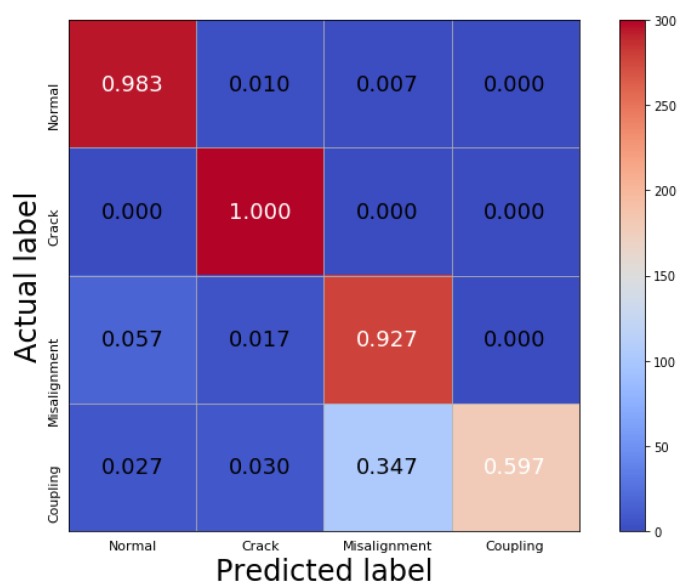
The confusion matrix with an SNR of −10 dB in Case 1.

In order to further study the recognition results under a strong noise background, the confusion matrix with an SNR of −10 dB is shown in Figure 6. The vertical coordinate denotes the actual labels and the horizontal coordinate denotes the predicted labels, the values on the main diagonal represent the correct recognition rate of the signals in each condition, and the other values represent the probability that the signals are misdiagnosed as other conditions. It can be seen that the network can perfectly identify the crack fault which is 100% correctly classified, and the healthy condition is almost fully detectable with a recognition rate of 98.3%. It also shows that the classification accuracy of the misalignment–crack coupling fault is only 59.7%, since it can be often misclassified as a shaft misalignment condition. This is possibly due to the fact that the effective feature information required to distinguish them is largely covered by the strong noise.

### 4.5. Case 2: Fault Diagnosis for Noise-Added Training Samples

#### 4.5.1. Results and Discussions

Differently from Case 1, this section directly adds Gaussian white noise of different SNR values to the raw measured signals to simulate the actual noisy environment. VMD and PPCA are used to realize noise reduction for noisy signals, and the designed optimized CNN is further used to complete the identification of crack and shaft misalignment for the rotor system.

Specifically, the ability of VMD to process nonstationary signals is first fully utilized to decompose the noisy signals into submodal functions with multiple frequency bands. However, [37,38,39,40,41,42,43] point out that the number of submodes *k* and penalty factor α in the algorithm have an obvious impact on the central frequency and bandwidth of the submodal function: a small value of *k* will lead to severe modal aliasing, while a large value of *k* will result in overdecomposition; and the effect of α on the bandwidth is that the larger the value, the smaller the bandwidth of the submodal function. The values of *k* and α are chosen as 10 and 10,000 respectively to fully decompose the noisy signals into multiple submodal functions with narrow frequency bands and effectively suppress the modal aliasing phenomenon. Then, 10 submodal signals are projected into 2 subspaces by the dimensionality reduction capability of PPCA to preserve the low-frequency information in the principal component subspace, and the signals of the remaining subspace are regarded as noise and discarded. Finally, the remaining denoised signals are randomly divided into a training set and a testing set, and then fault identification of crack and shaft misalignment for the rotor system is carried out by the optimized CNN. 

**Figure 7:**
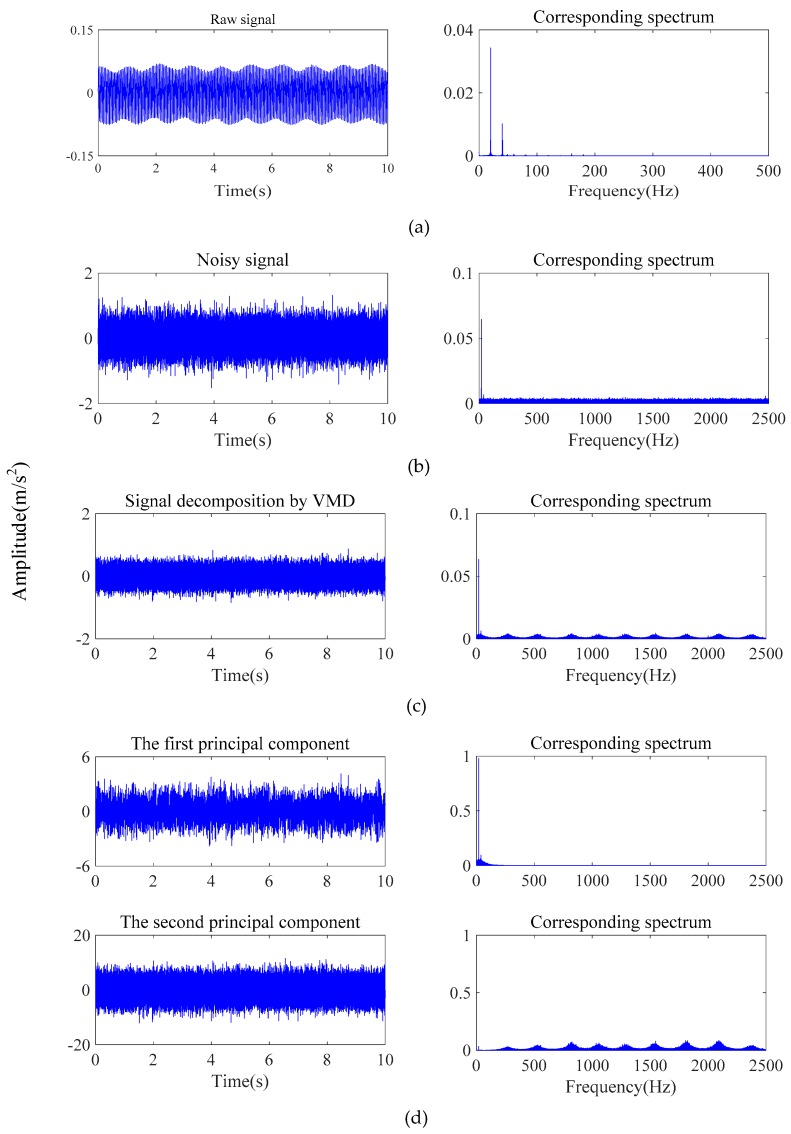
Signal processing: (**a**) raw signal; (**b**) signal with Gaussian white noise; (**c**) signal decomposition by VMD; (**d**) signal processed by PPCA.

**Figure 8:**
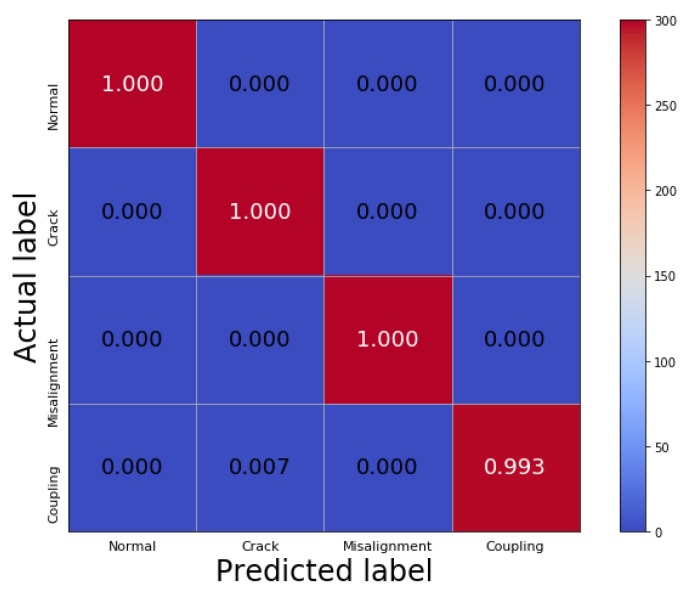
The confusion matrix with an SNR of −10 dB in Case 2.

Figure 7 shows the process of adding the raw vibration signals with Gaussian white noise, and signal decomposition by VMD and dimensionality reduction by PPCA. It can be seen that the high-frequency noise can be effectively eliminated, and the characteristics of the low-frequency signals are preserved after the processing by VMD and PPCA, which provides a basis for further utilizing CNN to achieve accurate fault classification. The figures in the right column also show that the corresponding spectrum amplitudes are significantly increased, which demonstrates that PPCA has the ability to enhance characteristic information in signals. Table 5 shows the testing accuracy based on the VMD-PPCA-CNN method with different SNR values. It can be seen that the proposed method can completely identify the different types of faults of crack and shaft misalignment in the rotor system under noisy environments. Figure 8 shows the corresponding confusion matrix with an SNR of −10 dB. Compared with the results in Case 1, it also demonstrates that the method can successfully distinguish all the conditions.

#### 4.5.2. Comparison of the Proposed Method and Other Methods

**Figure 9:**
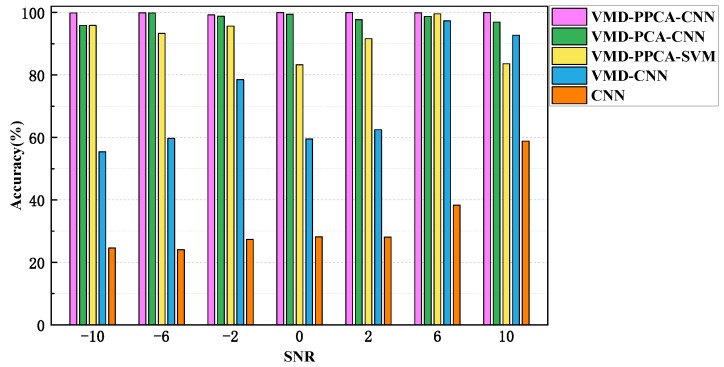
Results of the proposed method and other methods with different SNR values.

The fault identification results obtained with different methods are compared with the same dataset and different SNR values, including (1) VMD-PPCA-CNN, (2) VMD-PCA (principal component analysis)-CNN, (3) VMD-PPCA-SVM (support vector machine), (4) VMD-CNN, and (5) CNN. The parameters setting in VMD and CNN are consistent identical with those in the proposed method. In Method (2), the first principal component processed by PCA is directly inputted to the CNN; in Method (3), the SVM is used instead of the CNN as the classifier, and the polynomial kernel function is selected as the kernel function; in Method (4), 9 submodal signals with wider frequency bands are regarded as noise and discarded, and the remaining minimum frequency band signals are preserved and directly used as the original input of CNN. In Method (5), CNN is trained and verified directly with noisy signals. The average accuracy of 10 calculation results from different methods are shown in Figure 9.

As can be seen from Figure 9, the proposed method (Method (5)) achieves the highest recognition rates for fault identification of crack and shaft misalignment in the rotor system under different noisy environments. Several findings can be made for this comparison: The recognition rates using only CNN ares no more than 60% and are the lowest, indicating that the classification results are untrustworthy by CNN trained with noisy signals compared with the results in Table 4 for Case 1; Method (2) based on VMD-CNN can effectively increase the recognition rates, but compared with the Method (1) processed further by PPCA, its recognition rates are still low. At the same time, the method proposed in this paper has a consistent diagnosis performance for different SNR values, and in comparison with Method (2), PPCA has a more prominent noise reduction ability than traditional PCA. In Method (3), SVM is used to replace CNN as the feature classifier, and the resulting recognition rates are slightly lower than those of the proposed method, which shows that the fault diagnosis of the rotor system based on CNN can extract the deeper feature information from the vibration signals by deepening the network and, thus, the recognition performance of the network can be improved significantly.

#### 4.5.3. Verification Results at Different Rotating Speeds

**Figure 10:**
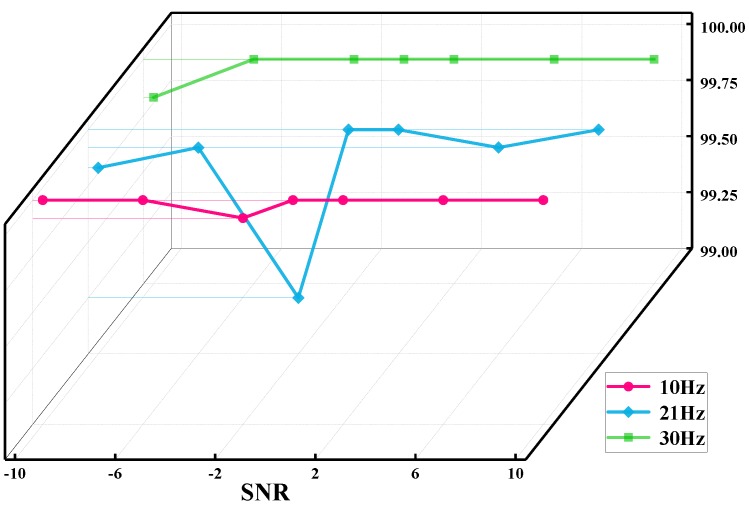
Comparison of recognition results with different rotating speeds and SNR values.

In order to discover the influence of rotating speeds on the recognition accuracy of the proposed method, the original measured vibration signals under the rotating speeds of 10 and 30 Hz are processed in the same way as the abovementioned 21 Hz signals, simulating the operating conditions of the rotor system at low, medium, and high speeds, respectively. Figure 10 illustrates the identification results at the three rotating speeds and different SNR values. It shows that the proposed method can achieve an overall recognition rate higher than 99.25%, and the recognition rates are close to 100% when the values of SNR are greater than 0. Particularly, the rotating speed has no obvious influence on the fault identification results.

#### 4.5.4. Visualization of Network Learning

**Figure 11:**
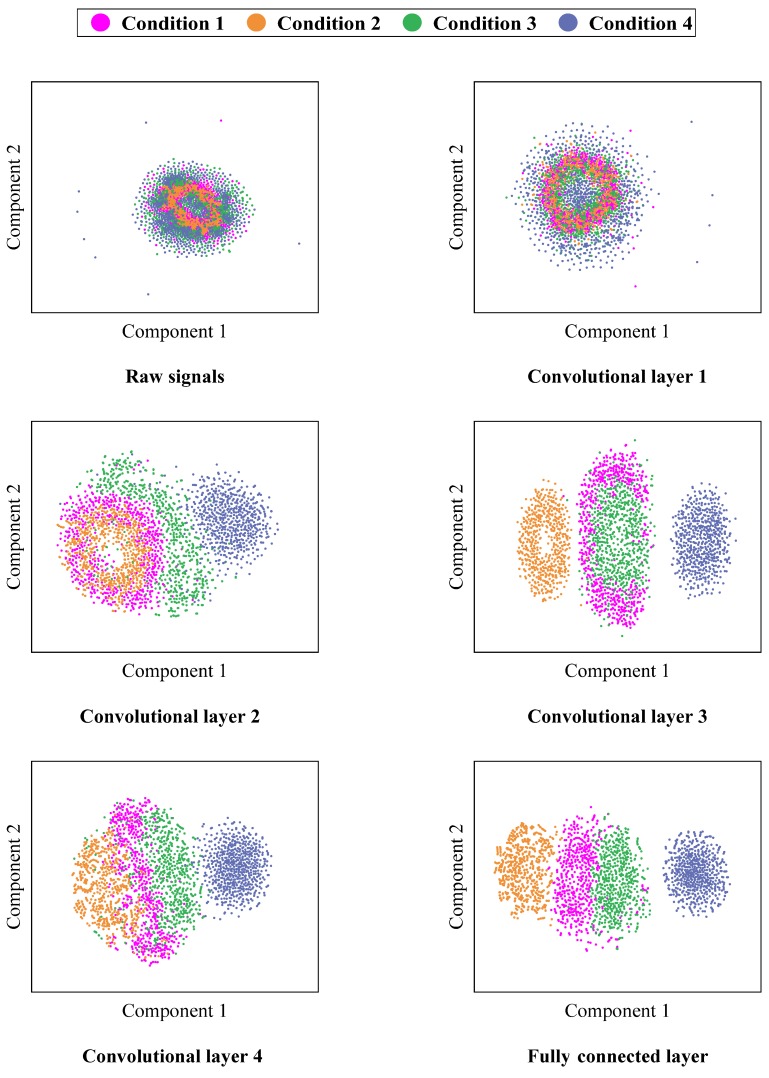
The visualization of features learned in different layers.

To illustrate the complex feature-learning process of the proposed CNN, the t-distributed stochastic neighbor embedding (t-SNE) algorithm [44] is adopted to reduce the high-dimensional features learned by each layer of CNN to a two-dimensional plane and visualize them with scatter plots. Taking the visualization of feature distribution of SNR = 10 dB signals at 21 Hz in the input layer, the four convolutional layers, and the fully connected layer as an example, the scatter plot of each layer is shown in Figure 11. It is clear that the samples of the four conditions in the input layer are clustered in the same space and distributed disorderly, so that they cannot be distinguished clearly; as the number of network layers increases, samples of different conditions are gradually separated, samples belonging to the same class are gathered together; and the samples in different conditions have been completely separated and clustered in different spaces at the fully connected layer with only individual samples overlapped. It also shows that the designed CNN has a good feature-learning ability, and can extract the intrinsic information of the input signals automatically by deepening the network, and ultimately achieve effective fault classification of the rotor vibration signals under different conditions through the output layer.

## 5. Conclusions

Focusing on the typical faults of crack and shaft misalignment in a rotor system under noisy environments, a method of fault identification is proposed, which combines variational mode decomposition (VMD) and probabilistic principal component analysis (PPCA) to denoise the collected vibration signals and then carry out a signal feature extraction and fault classification with an optimized convolutional neural network (CNN), and two experimental cases were considered to verify the validity and reliability of this method.

(1) The numbers of convolutional layers, kernels, fully connected layers, and nodes in the fully connected layers can be determined automatically by GA, reducing the manual adjustment process which often depends on expertise and is time-consuming. The optimized CNN has excellent domain adaptability and can effectively identify multitype faults in rotor systems.
(2) VMD and PPCA can not only remove the noise from vibration signals, but also preserve and amplify feature information in signals; combined with the optimized CNN, fault identification of crack and shaft misalignment for rotor systems under noisy environments can be effectively achieved.
(3) The recognition rates for the faults of crack and shaft misalignment in the tested rotor system with different SNR values and rotating speeds all reached more than 99%.

Compared with other methods, the proposed method is more effective and robust for removing the interference noise and extract the intrinsic features of the raw vibration signals, and can realize higher recognition accuracy for crack and shaft misalignment of rotor system under different degrees of noise interference, especially, since the recognition rate is still nearly 100% when the SNR value is −10. However, the hyperparameter optimization of CNN by GA is computationally intensive. Therefore, how to improve the performance of GA or find better algorithms for hyperparameter optimization in network requires further study.

## Figures and Tables

**Table 1:** Details of the rotor test bench.

Components	Type	Components	Type
Driving motor	SIEMENSE 1.5 kW	Coupling	Flexible coupling
Motor control system	VFD-M 1.5 kW	Bearing	SKF 6300
Eddy current displacement sensor	ZA21-0803	The material of rotary shaft	40Cr
Signal acquisition system	DHDAS5922N	The material of rotary disc	45# steel

**Table 2:** Parameters of genetic algorithm (GA).

Description	Symbol	Value
Population size	*N*	50
Maximum generation number	*n*	40
The probability of crossover	μ	0.95
The probability of mutation	υ	0.2
Maximum number of convolutional layers	$N_{C}$	5
Maximum number of fully connected layers	$N_{F}$	3
Maximum number of kernels in convolutional layers	$N_{K}$	64
Maximum number of nodes in fully connected layers	$N_{N}$	512

**Table 3:** Parameters of convolutional artificial neural network (CNN) optimized by GA.

Parameter	Value
The number of convolutional layers	4
The number of kernels in conv1	8
The number of kernels in conv2	16
The number of kernels in conv3	32
The number of kernels in conv4	32
The number of fully connected layers	1
The number of nodes in fully connected layer	128

**Table 4:** Testing accuracy with different SNR values.

SNR (dB)	−10	−6	−2	0	2	6	10
Accuracy (%)	87.67	99.83	100	100	100	100	100

**Table 5:** Testing accuracy with different SNR values.

SNR (dB)	−10	−6	−2	0	2	6	10
Accuracy (%)	99.83	99.92	99.25	100	100	99.92	100

## References

[1] Zhao J., DeSmidt H., Peng M. (2016). Harmonic transfer function based damage identification of breathing cracked Jeffcott rotor. Shock Vib..

[2] Bieryla D.J., Trethewey M.W., Lissenden C.J., Lebold M.S., Mitchell S., Maynard K.P. Shaft crack monitoring via torsional vibration analysis. Part 1–Laboratory tests. Proceedings of the 23nd International Modal Analysis Conference.

[3] Rahman A.G.A., Ismail Z., Noroozi S., Chao O.Z. (2013). Study of open crack in rotor shaft using changes in frequency response function phase. Int. J. Damage Mech..

[4] Tlaisi A., Akinturk A., Swamidas A.S.J., Haddara M.R. (2012). Crack detection in shaft using lateral and torsional vibration measurements and analyses. Turbomachinery.

[5] Ishida Y., Inoue T. (2006). Detection of a rotor crack using a harmonic excitation and nonlinear vibration analysis. J. Vib. Acoust..

[6] Mani G., Quinn D.D., Kasarda M.E.F. (2006). Active health monitoring in a rotating cracked shaft using active magnetic bearings as force actuators. J. Sound Vib..

[7] Mani G., Quinn D.D., Kasarda M.E.F. Structural health monitoring of rotor dynamic systems by wavelet analysis. Proceedings of the ASME International Mechanical Engineering Congress and Exposition, American Society of Mechanical Engineers.

[8] Chasalevris A.C., Papadopoulos C.A. Early detection of rotor cracks by measuring the coupled response under external excitation. Proceedings of the 8th IFToMM International Conference on Rotor Dynamics.

[9] Sinha J.K. (2007). Higher order spectra for crack and misalignment identification in the shaft of a rotating machine. Struct. Health Monit..

[10] Sinha J.K. (2009). Higher Order Coherences for fatigue crack detection. Eng. Struct..

[11] Guo D., Peng Z.K. (2007). Vibration analysis of a cracked rotor using Hilbert–Huang transform. Mech. Syst. Signal Process..

[12] Babu T.R., Srikanth S., Sekhar A.S. (2008). Hilbert–Huang transform for detection and monitoring of crack in a transient rotor. Mech. Syst. Signal Process..

[13] Nagaraju C., Rao K.N., Rao K.M. (2009). Application of 3D wavelet transforms for crack detection in rotor systems. Sadhana.

[14] Jesus G.M., Corral E., Castejon C., Garcia-Prada J.C. (2018). Effective crack detection in railway axles using vibration signals and WPT energy. Sensors.

[15] Saridakis K.M., Chasalevris A.C., Papadopoulos C.A., Dentsoras A.J. (2008). Applying neural networks, genetic algorithms and fuzzy logic for the identification of cracks in shafts by using coupled response measurements. Comput. Struct..

[16] Sinou J.J. (2008). Detection of cracks in rotor based on the 2× and 3× super-harmonic frequency components and the crack–unbalance interactions. Commun. Nonlinear Sci. Numer. Simul..

[17] Guo C.Z., Yan J.H., Yang W.C. (2017). Crack detection for a Jeffcott rotor with a transverse crack: An experimental investigation. Mech. Syst. Signal Process..

[18] Jun O., Eun H., Earmme Y., Lee C.-W. (1992). Modeling and vibration analysis of a simple rotor with a breathing crack. J. Sound Vib..

[19] Darpe A., Gupta K., Chawla A. (2004). Coupled bending, longitudinal and torsional vibrations of a cracked rotor. J. Sound Vib..

[20] Darpe A.K. (2007). Coupled vibrations of a rotor with slant crack. J. Sound Vib..

[21] Saavedra P.N., Cuitiño L.A. (2002). Vibration analysis of rotor for crack identification. J. Vib. Control.

[22] Prabhakar S., Sekhar A.S., Mohanty A.R. (2002). Crack versus coupling misalignment in a transient rotor system. J. Sound Vib..

[23] Patel T.H., Darpe A.K. (2009). Vibration response of misaligned rotors. J. Sound Vib..

[24] Sekhar A.S., Prabhu B.S. (1995). Effects of coupling misalignment on vibrations of rotating machinery. J. Sound Vib..

[25] Ioffe S., Szegedy C. Batch normalization: Accelerating deep network training by reducing internal covariate shift. Proceedings of the International Conference on Machine Learning.

[26] Dragomiretskiy K., Zosso D. (2014). Variational mode decomposition. IEEE Trans. Signal Process..

[27] Rockafellar R.T. (1973). A dual approach to solving nonlinear programming problems by unconstrained optimization. Math. Program.

[28] Tipping M.E., Bishop C.M. (1999). Probabilistic principal component analysis. J. R. Stat. Soc. Ser. B Stat. Methodol..

[29] Utsugi A., Kumagai T. (2001). Bayesian analysis of mixtures of factor analyzers. Neural Comput..

[30] Cardoso F.R., Achcar J.A., Piratelli C.L., Hermosilla J.L.G., Barbosa J.C. (2014). Bayesian analysis of employee suggestions in a food company. Int. J. Adv. Manuf. Technol..

[31] Dempster A.P., Laird N.M., Rubin D.B. (1977). Maximum likelihood from incomplete data via the EM algorithm. J. R. Stat. Soc. Ser. B Stat. Methodol..

[32] Jordan M.I., Jacobs R.A. (1994). Hierarchical mixtures of experts and the EM algorithm. Neural Comput..

[33] McLachlan G., Krishnan T. (2008). The EM Algorithm and Extensions.

[34] Zhang W., Peng G.L., Li C.H., Chen Y.H., Zhang Z.J. (2017). A new deep learning model for fault diagnosis with good anti-noise and domain adaptation ability on raw vibration signals. Sensors.

[35] Goyal P., Dollar P., Girshick R.B., Noordhuis P., Wesolowski L., Kyrola A. (2017). Accurate, Large Minibatch SGD: Training ImageNet in 1 Hour. arXiv.

[36] Keskar N.S., Mudigere D., Nocedal J., Smelyanskiy M., Tang P.T.P. On large-batch training for deep learning: Generalization gap and sharp minima. Proceedings of the 5th International Conference on Learning Representations.

[37] Wang Y.X., Markert R. (2016). Filter bank property of variational mode decomposition and its applications. IEEE Trans. Signal Process..

[38] Li J., Yao X., Wang H., Zhang J. (2019). Periodic impulses extraction based on improved adaptive VMD and sparse code shrinkage denoising and its application in rotating machinery fault diagnosis. Mech. Syst. Signal Process..

[39] Zhou C.J., Ma J., Wu J.D., Yuan X.Y. (2019). An adaptive VMD method based on improved GOA to extract early fault feature of rolling bearings. Int. J. Innov. Comput. Inf. Control.

[40] Yi C.C., Lv Y., Dang Z. (2016). A fault diagnosis scheme for rolling bearing based on particle swarm optimization in variational mode decomposition. Shock Vib..

[41] Wang X.B., Yang Z.X., Yan X.A. (2018). Novel particle swarm optimization-based variational mode decomposition method for the fault diagnosis of complex rotating machinery. IEEE-ASME Trans. Mech..

[42] Lian J.J., Liu Z., Wang H.J., Dong X.F. (2018). Adaptive variational mode decomposition method for signal processing based on mode characteristic. Mech. Syst. Signal Process..

[43] Li Z.P., Chen J.L., Zi Y.Y., Pan J. (2017). Independence-oriented VMD to identify fault feature for wheel set bearing fault diagnosis of high speed locomotive. Mech. Syst. Signal Process..

[44] Maaten L.V.D., Hinton G. (2008). Visualizing data using t-SNE. J. Mach. Learn. Res..

